# ATP Depletion Via Mitochondrial F_1_F_0_ Complex by Lethal Factor is an Early Event in *B. Anthracis*-Induced Sudden Cell Death

**DOI:** 10.4137/jcd.s2811

**Published:** 2009-08-27

**Authors:** Mitchell W. Woodberry, Leopoldo Aguilera-Aguirre, Attila Bacsi, Ashok K. Chopra, Alexander Kurosky, Johnny W. Peterson, Istvan Boldogh

**Affiliations:** 1 Medical Service Corps, Diagnostic System Division, United States Army Medical Research Institute of Infectious Diseases, Fort Detrick, Maryland 21702.; 2 Department of Microbiology and Immunology, University of Texas Medical Branch, Galveston, Texas, 77555.; 3 Department of Biochemistry and Molecular Biology, University of Texas Medical Branch, Galveston, Texas, 77555.

**Keywords:** anthrax lethal factor, mitochondria, F_1_F_0_ ATPase, pyroptosis

## Abstract

*Bacillus anthracis*’ primary virulence factor is a tripartite anthrax toxin consisting of edema factor (EF), lethal factor (LF) and protective antigen (PA). In complex with PA, EF and LF are internalized via receptor-mediated endocytosis. EF is a calmodulin-dependent adenylate cyclase that induces tissue edema. LF is a zinc-metalloprotease that cleaves members of mitogen-activated protein kinase kinases. Lethal toxin (LT: PA plus LF)-induced death of macrophages is primarily attributed to expression of the sensitive *Nalp1b* allele, inflammasome formation and activation of caspase-1, but early events that initiate these processes are unknown. Here we provide evidence that an early essential event in pyroptosis of alveolar macrophages is LF-mediated depletion of cellular ATP. The underlying mechanism involves interaction of LF with F_1_F_0_-complex gamma and beta subunits leading to increased ATPase activity in mitochondria. In support, mitochondrial DNA-depleted MH-S cells have decreased F_1_F_0_ ATPase activity due to the lack of F_0_6 and F_0_8 polypeptides and show increased resistance to LT. We conclude that ATP depletion is an important early event in LT-induced sudden cell death and its prevention increases survival of toxin-sensitive cells.

## Introduction

*Bacillus anthracis* is a gram-positive, aerobic, nonmotile, catalase-positive, rod-shaped, spore-forming bacterium that causes three forms of anthrax pathologies: cutaneous, inhalation, and gastrointestinal. Its major virulence factors are edema factor (EF), lethal factor (LF), and protective antigen (PA). In complex with receptor-binding moiety PA, EF and LF form toxins (edema toxin and lethal toxin, LT) that are internalized *via* receptor-mediated endocytosis.^1^,^2^ EF contains 767 amino acid residues and is a calmodulin-dependent adenylate cyclase that induces tissue edema. LF (776 amino acids) is a zinc-metalloprotease that cleaves mitogen-activated protein kinase kinases (MKKs).^3^

Recent data show that the susceptibility of murine macrophages to rapid LT killing is controlled by an extremely polymorphic gene, *Nalp1b*.^4^ Nalp1b (also known as NLRP1) is involved in the formation of a multimeric protein complex called inflammasome, which contains and activates caspase-1.^5^ Caspase-1 mediates the processing of inflammatory cytokines including IL-1β and IL-18.^6^ While many details of caspase-1 activation have been described, including the regulatory role of proteasomes in this process,^7^,^8^ it still remains to be shown how LT activates the inflammasomes and how caspase-1 activation leads to cell death. Macrophages, in which IL-1β and IL-18 had been deleted, display high susceptibility to caspase-1-mediated necrosis,^9^ indicating that these cytokines are associated with, but alone do not cause cell death.

LT-induced mitochondrial dysfunction is also a critical event in the cytolysis of murine macrophages.^10^ The rapid decline in mitochondrial function and changes in mitochondrial membrane potential in LT-treated cells indicate early damage to mitochondria.^10^ In support, two closely related mitochondrial proteins, Bcl-2/adenovirus E1B 19-kDa interacting protein 3 (Bnip3) and Bnip3-like (Bnip3L), were shown to be required for rapid cell death.^11^

It has been shown that LT-induced cell death is associated with ATP depletion.^10^ Oxidized ATP protects mice and cultured cells from LT toxicity by preventing endosome acidification, which is required for translocation of LF to the cytosol.^12^ This phenomenon appears to be associated with inhibition of endosome vacuolar H^+^-ATPase activity.^12^ An independent study showed that oxidized ATP also binds irreversibly to both α and β subunits of mitochondrial F_1_ ATPase and decreases its activity.^13^ Here we show that after internalization, LF localizes to mitochondria and interacts with F_1_F_0_ complex proteins including, subunits β and γ, resulting in increased ATPase activity and depletion of cellular ATP, which is a critical early event in LT-induced sudden (pyroptotic) cell death.

## Materials and Methods

### Cell cultures

Mouse alveolar macrophage (MH-S) cells (CRL-2019, American Type Culture Collection: ATCC; Manassas, VA) were cultured in RPMI 1640 medium. LA-4 (CCL-196, ATCC) mouse lung epithelial cells were cultured in F-12 Kaigh’s modified medium. All media were supplemented with 2 mM L-glutamine, 1.0 mM sodium pyruvate, 10% fetal bovine serum (FBS, Atlanta Biological, Lawrenceville, GA), 100 units/mL penicillin, and 100 μg/mL streptomycin. The cells were routinely subcultured using trypsin-EDTA and incubated under a humidified atmosphere of 95% air and 5% CO_2_ at 37 °C.

### Establishment of respiration-deficient cells

Mitochondrial DNA-deficient cells were developed as we described previously.^14^ MH-S cells were maintained in the presence of 200 ng/ml ethidium bromide for >60 population doublings. Respiration-deficient cells became pyrimidine auxotrophs and media were supplemented with uridine (50 μg/ml) and sodium pyruvate (120 μg/ml).^15^ Depletion of mitochondrial DNA (mtDNA) was confirmed by Southern blot hybridization.^16^ The DNA probe for hybridization was generated by PCR. The forward and reverse primer sequences were as follow: 5′-GCAGGAACAGGATGAACAGTCT-3′ and 5′-GTATCGTGAAGCACGATGTCAAGGGATGTAT-3′, respectively. The 725-bp product recognized a 10.8-kb restriction fragment when hybridized to MH-S mtDNA digested with *Bam*HI as described previously.^14^,^16^

### Annexin V assay

Cell death assays were performed as we previously described.^17^ Briefly, cells were treated with LT for 0, 1, 1.5, 2, 2.5, 3, 4, and 6 h, then washed with phosphate buffered saline (PBS), resuspended in 1X binding buffer (0.1 M HEPES/NaOH, pH 7.4, 1.4 M NaCl, 25 mM CaCl_2_) stained for 30 min with Annexin V-Phycoerythrin (PE) and 7-Aminoactinomycin D (7-AAD) (Annexin V-PE Apoptosis Detection Kit I, Becton Dickinson, San Jose, CA). Changes in fluorescence were analyzed by flow-cytometry (BD FACSCanto™ Becton Dickinson). A minimum of 15,000 cells were collected and analyzed using BD FACSDiva™ software (Becton Dickinson).

### Microscopic analysis of morphological changes

Cells on cover-slips were LT-exposed (250 ng/ml PA + 50 ng/ml LF), fixed in formaldehyde and stained to visualize mitochondria with MitoTracker red (MTr, 50 nM, Molecular Probes Inc, Eugene, OR) and the DNA with SYBR green I (1:1,000 dilution, Molecular Probes). Confocal microscopy was performed using a Zeiss LSM510 META system [488 nm for excitation of SYBR green I (green) and 543 nm excitation of MTr (red)]. Images were superimposed using MetaMorph software Version 6.0r9.

### Assessment of ATP levels

ATP levels were determined as described previously.^18^ Briefly, ATP from mock- and LT-treated cells was released by boiling in distilled water (for 3 min). The lysates were then centrifuged at 12,000 × g for 5 min at 4 °C. ATP levels in supernatants were measured using the ATP Determination Kit (Molecular Probes). Luminescence was determined in a Veritas Microplate Luminometer (Turner Biosystems, Sunnyvale, CA).

### Assessment of poly (ADP-ribose) polymerase-1 (PARP) activity

The cells (1 × 10^7^) were centrifuged at 2,000 × g (Microcentrifuge 1236V, Centronix) for 5 min at room temperature. The pellet was then resuspended in 10 pellet volumes of 50 mM Tris, pH 8.0, 25 mM MgCl_2_, and 0.1 mM phenylmethanesulfonyl fluoride (PMSF). The suspensions were transferred to chilled microcentrifuge tubes and sonicated (Model GEX 130, Ultrasonic Processor) 3-times for 10 s at a time. Subsequently, the disrupted cells were centrifuged at 3000 × g (Silent SPIN, Continental Lab Products) for 5 min at 4 °C to remove insoluble material. The protein concentration of the supernatant was then measured using the Bio-Rad Protein Assay. Twenty micrograms of protein were used to determine PARP activity using the Trevigen PARP assay kit (Trevigen, Helgerman CT) according to the manufacturer recommendations. The 3H-mediated radioactivities were determined in a liquid scintillation counter (LS 6000IC, Beckman, Fullerton, CA).

### Measurement of mitochondrial membrane potential (mΔΨ)

Cells were loaded in culture medium with 5,5′,6, 6′-tetrachloro-1,1′,3,3′-tetraethylbenzimidazol-carbocyanine iodide (2 μM final concentration; JC-1; Invitrogen, Carlsbad, CA) for 15 min.^14^ The cells were then analyzed by flow cytometry (488 nm, 535/585 nm excitation and emission, respectively) using a BD FACSCanto™ Flow Cytometer. A minimum of 15,000 cells per sample was analyzed using FACSDiva™ software. To depolarize the mitochondrial membrane, carbonyl cyanide 3-chlorophenylhydrazone (CCCP, Invitrogen) was used at a final concentration of 50 μM.

### Assessment of Mitochondrial Permeability Transition Pore (MPTP) opening

Cells were loaded in culture medium with 1 μM acetoxymethyl ester of calcein (calcein AM, Molecular Probes) for 15 min at 37 °C^19^ and 50 nM MitoTraker Red (Molelcular Probes Inc) was added to co-stain mitochondria. The cytosolic calcein signal was quenched by CoCl_2_ (100 μM), as described previously.^19^ Fluorescence was visualized by microscopy (Zeiss LSM510 META System) at 488 and 525 nm excitation and emission wavelengths, respectively.

### Measurement of intracellular Reactive Oxygen Species (ROS) levels

2′,7′-dichlorodihydro-fluorescein diacetate (H_2_DCF-DA; Molecular Probes) was used to determine changes in cellular ROS levels.^17^ H_2_DCF-DA is a redox-sensitive cell-permeant dye, which is nonfluorescent until removal of the acetate groups by intracellular esterases and oxidation occurs by reactive oxygen species. Briefly, cells were treated with increasing doses of LT [1000 ng/ml (800 ng/ml PA + 200 ng/ml LF); 500 ng/ml (400 ng/ml PA + 100 ng/ml LF); 250 ng/ml (200 ng/ml PA + 50 ng/ml LF); 125 ng/ml (100 ng/ml PA + 25 ng/ml LF)] and loaded with H_2_DCF-DA at 5 μM final concentration for 15 min at 37 °C. After washing, changes in DCF fluorescence of cells were determined by FACSCanto™ at time points when cells showed >95% viability. The mean fluorescence of 15,000 cells was determined in independent experiments (n =4–7). To confirm results, cells at 70% confluence were loaded with 50 μM H_2_DCF-DA on 24-well plates (Costar, Corning, NY). Changes in fluorescence intensity in mock-treated, LT-treated, and control cells were measured using an FL×800 Microplate Fluorescent Reader (Bio-Tek Instruments, Winooski, VE) at 488 nm excitation and 530 nm emission wavelengths.

### Mitochondria isolation and complex activity assessment

Cell pellets were incubated in 10X volume of hypotonic buffer [10 mM KCl, 20 mM 4-morpholinopropanesulfonic acid (MOPS), and 1 mM ethylene glycol-bis (β-aminoethyl ether)-N,N,N′, N′-tetraacetic acid (EGTA)] for 20 min then Dounce-homogenized. The homogenate was clarified at 800 × *g* and the supernatants were centrifuged at 10,000 × *g* to collect mitochondria. Mitochondrial pellets were washed and resuspended in 10 mM KCl, 20 mM MOPS, and 1 mM EGTA containing 200 mM sucrose and 50 mM mannitol. In selected experiments, fresh mitochondrial suspensions were purified on a continuous sucrose gradient (0.25 M to 1.5 M). The oxygen consumption rates of mitochondria were determined at 30 °C with a Clark-type oxygen electrode (Strathkelvin Oxygen System Model 782, Strathkelvin Instruments, United Kingdom) as we previously described.^14^ Respiratory complex (I, II, III and IV and complexes I +III) activity measurements were undertaken as described previously.^14^,^20^ Activities were normalized to succinate dehydrogenase (SDH) activity.^14^,^21^

### Isolation and fractionation of mitochondrial complex proteins

Blue native polyacrylamide gel electrophoresis (BN-PAGE, 4%–12%) was performed essentially as described previously.^22^ Electrophoresis was run at 250 V for the first 30 min at 4 °C and then at a constant current of 5 mA. Electrophoresis was stopped when the tracking line of Coomassie Brilliant Blue G-250 dye (Sigma Aldrich, St. Loins, MO) left the bottom of the gel.^22^ Mitochondrial complexes were excised from blue native gels and placed into wells of a 10% sodium dodecyl sulfate (SDS)-polyacrylamide gel for separation of individual complex proteins.

### Far-Western Blot analysis

Proteins were separated by SDS-polyacrylamide gel electrophoresis (PAGE), transferred to a nitrocellulose membrane (Schleicher and Schuell BioScience, Keene, NH), treated with 6 M guanidine-HCl (in PBS), and then re-natured with successive dilutions of guanidine-HCl and 1 mM dithiothreitol (DTT), as we previously described.^23^ After blocking with 5% nonfat dry milk in blocking buffer (PBS, 0.5% Tween 20), the membranes were incubated with LF (250 ng/ml) or PA (250 ng/ml) in blocking buffer for 3 h at 4 °C. Binding of LF or PA was detected by anti-LF and anti-PA antibodies (Advanced ImmunoChemical, Inc., Long Beach, CA). After overnight incubation at 4 °C and extensive wash, detection was performed by enhanced chemiluminescence (GE Healthcare Bio-Sciences, Piscataway, NJ) and signals were visualized by autoradiography.

### Protein sequencing

Automated Edman N-terminal microsequencing of excised stained bands was carried out with an Applied Biosystems cLC 494 Protein Sequencer (Foster City, CA), as we previously reported.^24^ Proteins were identified using the BLAST search program and National Center for Biotechnology Information (NCBI) as well as Swiss-Prot databases. N-terminal-blocked proteins were identified by mass spectrometry. Briefly, after staining with Coomassie Blue, bands were excised and subjected to trypsin digestion. Mass spectra of peptide digests were obtained using a Model 4800 MALDI-TOF-TOF MS (Applied Biosystems, Foster City, CA). Proteins were identified using the Swiss-Prot database and the Mascot algorithm as we reported previously.^24^ MS analyses and protein sequencing were conducted by the Biomolecular Resource Facility at UTMB.

### Immunoprecipitation

Cells were exposed to LT (500 ng/ml PA and 100 ng/ml LF) for 30 min. Mitochondria were isolated, purified on a sucrose gradient (0.25 M to 1.5 M) and lysed in modified RIPA buffer (50 mM Tris-HCl, pH 7.4, 150 mM NaCl, 1 mM EDTA, 0.25% sodium deoxycholate, 1% Nonidet P-40, 1 mM PMSF, 1 mM NaF, 1 mM Na_3_VO_4_, and 1 μg/ml each of aprotinin, leupeptin, and pepstatin).^25^ Extracts were pre-cleared with protein A-Sepharose 4B (Sigma) for 10 min at 4 °C and the cleared lysate incubated with anti-LF or anti-PA antibody (Advanced ImmunoChemical, Inc.) for 3 h at 4 °C. Immune complexes were captured by adding 30 μl of protein A-Sepharose beads (Thermo Scientific Life Science Research, Rockford, IL) for 3 h at 4 °C. Beads were washed three times with cold PBS. Immune complexes eluted by incubation in loading buffer were fractionated by 10% SDS- PAGE and then analyzed by Western blotting.

### Western Blot analysis

Equal amounts of protein from cell lysates were electrophoresed on 10% SDS-PAGE.^17^ Fractionated proteins were transferred onto nitrocellulose membranes (Schleicher and Schuell BioScience, Keene, NH). The membranes were blocked with 5% nonfat dry milk in TBS-T (20 mM Tris-HCl, pH 8.0, 125 mM NaCl with 0.025% Tween 20) overnight and then incubated with primary antibody to F_1_F_0_ α subunit (1:500; BD Biosciences, San Jose, CA), to β subunit (1:1000; Molecular Probes), to LF and to PA (Advanced ImmunoChemical, Inc.) for overnight at 4 °C. At the end of the incubation, the membrane was washed in TBS-T and subsequently incubated with horse radish-peroxidase-conjugated secondary antibody (GE Healthcare Bio-Sciences; 1:3000 dilution) in TBS-T for 1 h. After washing, detection was performed by enhanced chemiluminescence (GE Healthcare Bio-Sciences).

### Assessment of complex V (F_1_F_0_-ATPase) activity

Purified mitochondria were sonicated for a total of 3 × 30-s bursts on ice. One hundred μg of sub-mitochondrial particles in 50 mM HEPES-KOH (pH 8.0), 1 mM MgCl_2_, and 250 mM sucrose were added to a cuvette containing 25 U pyruvate kinase, 24 U lactate dehydrogenase, 20 μM rotenone (to inhibit complex I), 0.74 μM antimycin A (to inhibit complex III), 5 mM phosphoenolpyruvate, and 175 μM NADH. The reaction was initiated upon addition of 2 mM ATP (final concentration). The assays were performed in the presence or absence of the mitochondrial ATPase inhibitors (15 μM oligomycin or 60 μM aurovertin B) to estimate the percentage of ATPase activity that was related to the F_1_ or F_0_F_1_ complex in mitochondria.^26^ Changes in absorption at room temperature were measured spectrophotometrically (DU^®^ 530 Life Science UV/VIS spectrophotometer, Beckman).^26^

### Reagents

All reagents were purchased from Sigma-Aldrich unless otherwise stated.

### Statistical analysis

Results were analyzed for significant differences using analysis of variance (ANOVA) procedures and Student’s t-tests (Sigma Plot 6.0). Data are expressed as the mean ± SE. Results were considered significant at *p* < 0.05. (*p < 0.05, **p < 0.01, ***p < 0.001, ****p < 0.0001).

## Results

### Depletion of ATP from LT-treated cells

**Figure 1:**
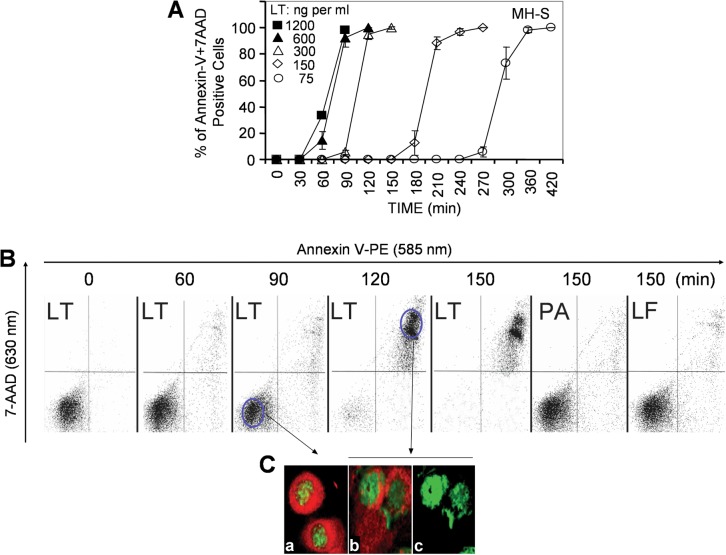
Sudden death of LT-treated MH-S macrophages. **A**) Concentration-independent pyroptosis of MH-S cells shown by Annexin V-PE and 7AAD-staining. Changes in cell-associated fluorescence intensities were determined by flow cytometry. LT: 1200 ng/ml (1000 ng/ml PA + 200 ng/ml LF); 600 ng/ml (500 ng/ml PA + 100 ng/ml LF); 300 ng/ml (200 ng/ml PA + 50 ng/ml LF); 150 ng/ml (125 ng/ml PA + 25 ng/ml LF); 75 ng/ml (62.5 ng/ml PA + 12.5 ng/ml LF). **B**) Kinetic changes in Annexin V binding and 7-AAD uptake in MH-S cells exposed to LT (250 ng/ml PA + 50 ng/ml LF). **C**) LT-induced morphological changes of MH-S cells. Cells on cover-slips were LT-exposed and stained to visualize mitochondria with MitoTracker red (MTr) and the DNA with SYBR green I. **A**) and **B**) are superimpositions of MTr and SYBR green I-stained images of cells **C**) SYBR green I-stained image of b. Magnification: 134×.

**Figure 2:**
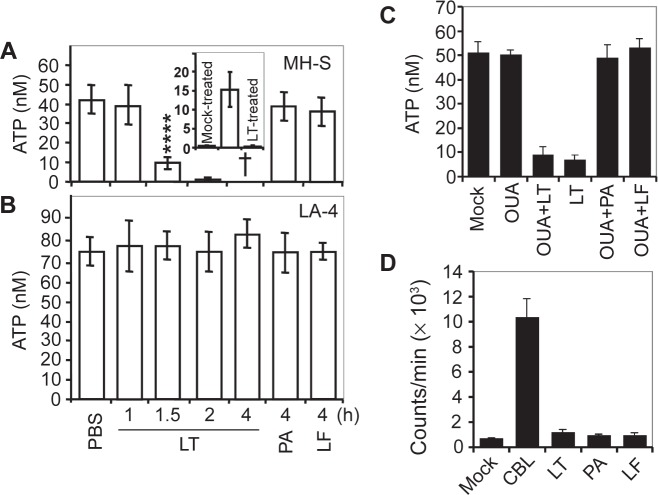
LT depletes cellular ATP in MH-S but not in LA-4 cells. **A**) MH-S cells were treated with LT (PA 250 ng/ml + LF 50 ng/ml) and cellular ATP levels were determined by bioluminescence assays. PA (250 ng/ml) or LF (50 ng/ml) alone had no effect on ATP levels. Inset: Lack of ATP in extracellular milieu at 2 hr after LT addition. Middle column, ATP released by sonication from mock-treated cells. **B**) ATP levels in LA-4 cells were not affected by LT. Cells were treated and ATP levels were determined as in A. In panels A and B, ATP levels were normalized for protein concentrations of individual samples. Data are ± SEM from 6 to 8 experiments run in triplicate. ^†^Denotes that more than 95% of cells were killed. ****p < 0.0001. **C**) Inhibition of Na^+^/K^+^-ATPase did not alter LT-induced changes in ATP levels. MH-S cells ± ouabain (OUA) (1 mM) and ± LT (250 ng/ml PA + 50 ng/ml LF) were harvested and ATP levels were determined at 90 min after treatment. **D**) PARP is not activated during LT-induced cell killing. Cells were treated with LT (250 ng/ml PA and 50 ng/ml LF) and harvested for 90 min to determine PARP-1 activity. Data are averages of three independent experiments. In controls, cells were treated with 50 μM chlorambucil (CBL).^57^

High or low doses of LT induced a simultaneous Annexin-V binding and loss of membrane integrity resembling a sudden type of cell death (pyroptosis) in murine macrophages (MH-S cells, low passage) (Figs. 1A, B and C). Next, we investigated the changes in intracellular ATP levels as possible early consequences of LT treatment.^10^ At 1 h there were no changes, while LT treatment decreased ATP levels by 80% at 1.5 h in MH-S cells (Fig. 2A). In cells exposed to lower concentrations of LT, intracellular ATP levels were depleted at a proportionally slower rate (data not shown). There were no changes in intracellular ATP levels in LT-resistant LA-4 cells after exposure to toxin (Fig. 2B). To exclude loss of ATP *via* cytoplasmic membrane, we show that there was no detectable ATP in the cell culture supernatant of LT-treated cells (Fig. 2A inset). In addition, we show that ouabain (1 mM; inhibits Na^+^/K^+^-ATPase)^27^ did not prevent or delay depletion of ATP from LT-treated cells (Fig. 2C). Our results also showed that in LT-treated cells, the activity of poly(ADP-ribose) polymerase-1 (PARP-1), which can cause the depletion of cellular energy stores,^28^ was not significantly changed (Fig. 2D).

**Figure 3:**
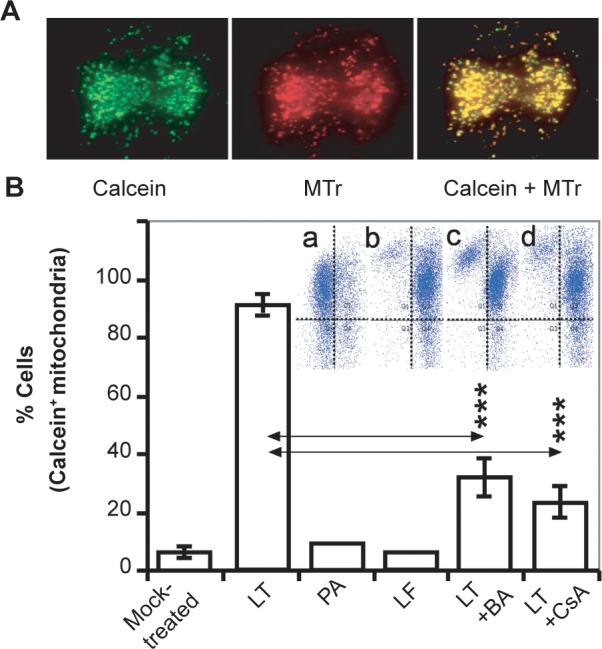
LT induces opening of MPTP. **A**) Mitochondrial uptake of calcein in LT-treated cells. Cells on cover slips were exposed to LT (250 ng/ml PA + 50 ng/ml LF) and at 60 min cells were loaded with calcein-AM (green) and stained with MitoTracker Red. Green and red images were superimpositioned using MetaMorph software. Magnification: 190×. **B**) Opening of MPTP was only partially prevented by cyclosporin A (CsA) or bongkrekic acid (BA). Cells were treated with BA (10 μM) or CsA (5 μM) for 15 min and LT was added. The percentage of cells showing calcein-mediated fluorescence was evaluated at ∼75 min thereafter. Data are ± SEM from 3 to 4 experiments run in triplicate. ***p < 0.001. Inset: BA or CsA had no insignificant effect on dissipation of mΔΨ. **A**) mock-treated cells; **B**) LT-exposed cells; **C**) LT-exposed cells treated with BA d, LT-exposed cells treated with CsA.

The mitochondrial permeability transition pore (MPTP) opening plays a key role in cell death, impacting mitochondrial membrane potential (mΔΨ) and ATP levels.^29^,^30^ We show that in LT-exposed MH-S cells (but not LT-treated LA-4 cells or MH-S cells treated with LF or PA alone) calcein-AM (2 μM, an indicator of MPTP opening)-mediated fluorescence co-localized with MitoTracker Red, indicating calcein uptake by mitochondria at 75 min after exposure (Fig. 3A). Inhibitors of MPTP opening, such as cyclosporin A (CsA; 5 μM) and bongkrekic acid (BA, 10 μM), decreased calcein-AM-derived fluorescence in mitochondria of LT-treated cells (Fig. 3B). However, CsA or BA did not protect cells from LT-mediated dissipation of mΔΨ (Fig. 3B, inset) and ATP depletion as well as sudden cell death (data not shown). These results indicate that MPTP opening is a consequence of ATP depletion and it is not directly related to the death of LT-exposed cells.

### LF interacts with mitochondrial proteins

**Figure 4:**
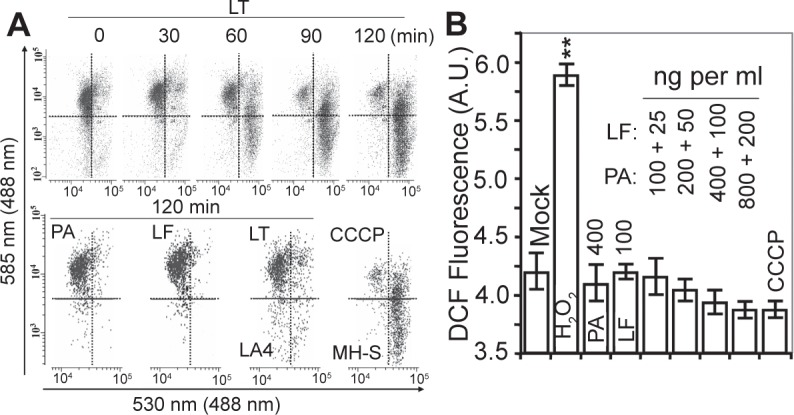
Decrease in mΔΨ and intracellular ROS levels after LT treatment. **A**) Dissipation of mΔΨ in LT-treated cells. MH-S cells were exposed to LT (250 ng/ml PA + 50 ng/ml LF) and at 0, 30, 60, 90, and 120 min thereafter cells were loaded with JC-1 (10 min, at 37 °C, 1 μM final concentration). Upper row shows the results of a typical set of experiments out of five. Lower panels show controls, including cells treated with PA (250 ng/ml) or LF alone (50 ng/ml) for 120 min. LT did not change mΔΨ in LA-4 cells. CCCP (2 μM) dissipates mΔΨ within 15 min. The y-axis represents energized mitochondria that have retained the JC-1 aggregates [red; 585 nm (488 nm)]. A decrease in red (or increase in green) fluorescence represents de-energized mitochondria [green; 530 nm (488 nm)]. **B**) LT treatment decreases intracellular levels of ROS. MH-S cells were treated with increasing concentrations of LT, PA or LF as indicated, and then cells were loaded with H_2_DCF-DA for 15 min. H_2_O_2_ treatment was used as a positive control. The changes in fluorescence intensities were assessed in an FLx800 microplate reader. Results are expressed as means ± SEM values of independent experiments (n = 5–9). A.U., arbitrary units. **p < 0.01.

Mitochondrial membrane potential is maintained by respiratory complexes and F_1_F_0_ complex, the latter utilizes large quantities of ATP.^31^,^32^ A representative set of flow-cytometry histograms showed that LT dissipated mΔΨ in >90% of cells by 90 min of LT addition. In control experiments PA or LF alone had no effect, while CCCP, a mitochondrial membrane potential uncoupler, eliminated mΔΨ (Fig. 4A). Intracellular ROS levels either remained unaffected or decreased in response to LT exposure, which is consistent with the dissipation of mΔΨ (Fig. 4B). A correlation between ATP depletion and loss of mΔΨ shortly after LT treatment suggested a direct interaction between LF and mitochondrial inner membrane complexes.

**Figure 5:**
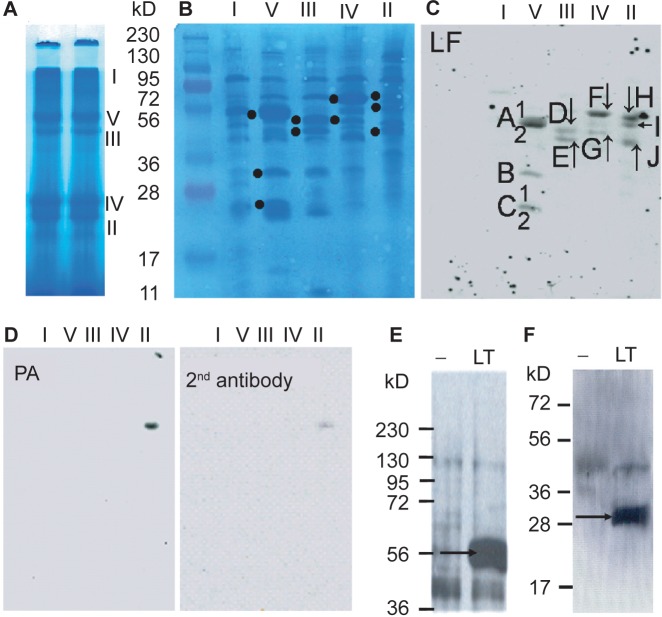
LF but not PA binds to respiratory complex and F_1_F_0_ complex proteins. MH-S cells were propagated to large volumes and mitochondria were isolated, purified by sucrose gradient and lysed as in Materials and Methods. **A**) Mitochondrial complexes were separated using Blue Native-polyacrylamide gel electrophoresis. B, Complex proteins were resolved on an SDS-PAGE, transferred to a PVDF membrane and protein bands were visualized by Coomassie blue-staining. **^•^**Protein bands that interact with LF (panel B) were subjected to identification by sequencing. C, Proteins were transferred to PVDF membrane and LF interacting respiratory complex (I, II, III, IV) and F_1_F_0_ complex (complex V) proteins were visualized by Far-Western blot analysis. Proteins (Table 1) were identified by Edman N-terminal sequencing (or MALDI-TOF/TOF MS). D, Full-length PA does not interact with respiratory complex or F_1_F_0_ subunit proteins. E and F, LF interacts with subunit γ and subunit β of the F_1_F_0_ complex *in vivo.* Cells were mock-treated (−) or exposed to LT (500 ng/ml PA + 100 ng/ml LF), and mitochondria were isolated and purified. Mitochondrial lysates were immunoprecipitated with anti-LF Ab and reacted with Ab to subunit-β (E) and subunit-γ (F) of F_1_F_0_ complex. Antibody binding was detected by the enhanced chemiluminescence assay and visualized by autoradiography.

To test this possibility, mitochondrial complexes from MH-S cells were separated by BN-PAGE and individual complexes were fractionated on SDS-PAGE (Figs. 5A, B). Using Far-Western blot approaches, we show interactions between LF and ATP synthase (F_1_F_0_ complex), as well as respiratory complex proteins (Fig. 5C). LF-interacting proteins were identified as described in Materials and Methods. Results in Figure 5C (and those summarized in Table 1) show that LF interacts with the F_1_F_0_ complex γ polypeptide (H^+^ transporting mitochondrial F_1_ complex, Fig. 5C, band B), F_1_F_0_ complex subunit β (H^+^ transporting mitochondrial F_1_ complex, Fig. 5C, band A1), and another three precursors of the F_1_F_0_ complex (Fig. 5C, bands A2, C1, C2, Table 1). In addition, LF binds to precursors of ubiquinol-cytochrome b-c1 complex subunit 1, ubiquinol-cytochrome b-c1 complex subunit 2, structural proteins of complex III, as well as to a precursor form of NADH dehydrogenase (ubiquinone) flavoprotein 1 (NDUFV1) in complex I (NADH:ubiquinone oxidoreductase) (Fig. 5C, Table 1). There were additional interactions of LF with trifunctional enzyme subunit beta precursor, 3-oxoacid CoA transferase, precursor protein of short-chain specific acyl-CoA dehydrogenase (citric acid cycle) and HSP60 (Fig. 5C, Table 1). It is noteworthy that the precursor protein NDUFV1 (complex I) co-migrated with complex II. Similarly, citric acid cycle proteins were found to be associated with respiratory complexes II and IV (Fig. 5C, Table 1). These observations are not surprising since mitochondria were solubilized and complexes were separated under mild nonionic detergent conditions.^22^ The association of respiratory complex proteins (supercomplexes; e.g. I + III, and I + III + IV) is well-established in mammalian mitochondria.^33^

The full-length (83 kD) (Fig. 5D) or truncated (63 kD) (data not shown) form of PA showed no reactivity with any of the respiratory or F_1_F_0_ complex proteins. The band seen on Figure 5D is a non-specific band as it was detected by the secondary antibody alone as well. Using Far-Western blot approaches, we observed similar interactions of LF between proteins of mitochondrial respiratory complexes and F_1_F_0_ complex from LA-4 cells (data not shown).

To test whether LF can interact with mitochondrial proteins *in vivo*, cells were treated with LT for 90 min and mitochondria were isolated, purified and lysates were prepared. The protein-protein interactions were analyzed by immunoprecipitation using anti-LF antibody. The precipitated proteins were identified by Western blot analysis using specific antibodies to γ (Fig. 5E) and β (Fig. 5F) polypeptides of F_1_F_0_ complex. Our result indicates that LF interacts with both β and γ subunits of F_1_F_0_ complex in MH-S cells. Because precursor proteins are not functional, we did not test them. Although we observed extensive interactions between LF and mitochondrial proteins of LA-4 cells in Far-Western analysis, we were not able to pull-down protein complexes with anti-LF antibody (data not shown), suggesting that no interaction with γ and β polypeptides of F_1_F_0_ complex takes place in LA-4 cells.

### Increased ATPase activity in LT-treated cells

**Figure 6:**
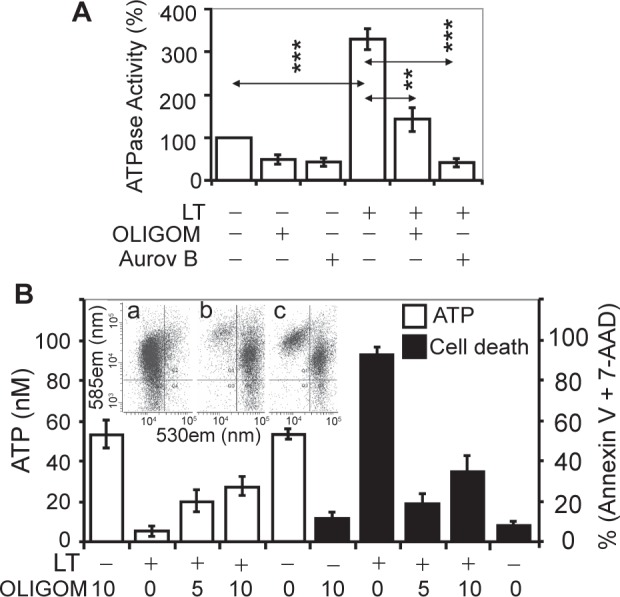
F_1_F_0_ inhibitors decrease ATP depletion from LT-exposed MH-S cells. **A**) LT increases ATPase activity in MH-S cells. Parallel cultures of cells were mock-, oligomycin (10 μM, OLIGOM)- or aurovertin B (60 μM, Aurov B)-treated and 30 min thereafter LT exposed for 90 min. Mitochondria were isolated and lysed, and ATPase activity was determined as in Materials and Methods. **B**) empty columns; F_1_F_0_ inhibitor oligomycin delays ATP depletion from LT-exposed MH-S cells. Parallel cultures of cells were oligomycin (5 and 10 μM)-treated and LT exposed. ATP levels were determined as in Figure 2A and B. B, filled columns; Olygomycin increases viability as determined at 120 min post-exposure by Annexin V-7-AAD assay. Data are ± SEM from 3 to 4 experiments run in triplicate. **p < 0.01, ***p < 0.001. Inset: Oligomycin partially prevents loss of mΔΨ. a, mock-treated; b, LT-exposed; c, oligomycin (10 μM)- and LT-exposed.

Our results revealed no changes in respiratory complex I, II, III, and IV or coupled activities of I and III, as well as II and III of mitochondria isolated from LT-exposed cells compared to unexposed controls (data not shown). These results suggest that proton pumping by respiratory complexes may not be impaired in LT-treated cells. Interactions between LF and β and γ polypeptides of F_1_F_0_ complex, rapid depletion of ATP, as well as the sudden cell death raised the possibility that LF increases ATPase activity. The ATPase activity of complex V was assessed using mitochondrial homogenates prepared at 90 min after LT addition. Figure 6A shows that ATPase activity in mitochondrial homogenates from LT-treated MH-S cells was increased compared to the mock-treated control. Oligomycin (10 μM), which binds to F_0_, 34,35 inhibited the ATPase activity of mitochondria from mock- and LT-treated cells by 52% and 45%, respectively (Fig. 6A). In support aurovertin (60 μM), which binds to catalytic β-subunits in the F_1_-ATPase,^36^,^37^ decreased ATP hydrolysis more efficiently, 55% vs. 79% in mitochondria from mock- and LT-treated cells, respectively (Fig. 6A).

Oligomycin treatment resulted in partial protection from LT-induced ATP depletion (Fig. 6B) and increased survival as well as the percentage of cells showing physiological mΔΨ (Fig. 6B inset). For example, 37% ± 7% of oligomycin (10 μM)-treated cells retained mΔΨ (Fig. 6B, inset c) and ATP levels were decreased to 28 ± 4.8 nM from 53 ± 6.8 nM. Without oligomycin, >95% of cells lost their mΔΨ (Fig. 6B, inset b) and ATP levels were 4.8 ± 2.6 nM (from 53 ± 6.8 nM) determined at 1.5 h after LT addition. Cell viability determined at 2 h exposure correlated well with the decreased ATP levels (Fig. 6B, filled columns). Aurovertin B (even at 2.5 μM) severely affected survival of MH-S cells, and consequently we were unable to obtain meaningful results. Collectively, these results suggested that an increase in F_1_F_0_ ATPase activity contributed to LT-mediated ATP depletion, thereby steering cells toward sudden cell death.

### Increased resistance of *ρ*^0^MH-S cells to LT

**Figure 7:**
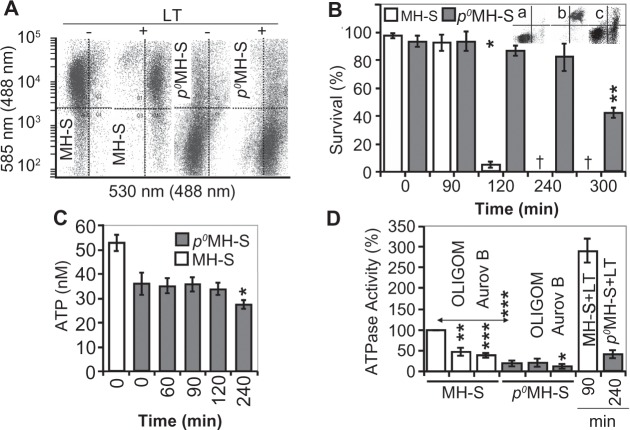
*p*^0^MHS cells show increased resistance to LT. **A**) *p*^0^MHS cells show a lower mΔΨ compared to MH-S cells. Parallel cultures of MHS and *p*^0^MHS cells were LT-treated and 90 min thereafter loaded with JC-1 (1 μM final concentration). Changes in JC-1 fluorescence were assessed by flow cytometry as in the legend to Figure 3. **B**) *p*^0^MHS cells exhibit increased resistance to LT. Parallel cultures of MH-S and *p*^0^MHS cells were LT-exposed and the percentage of Annexin V-7-AAD positive cells were determined. Inset: representative histograms of mock-treated **A**) LT-exposed MH-S **B**) and LT-exposed *p*^0^MH-S **c**) cells at 120 min after toxin addition. **C**) Delayed ATP depletion in *p*^0^MH-S cells after LT addition. Intracellular ATP levels were determined as in Materials and Methods. ^†^Denotes that more than 95% of cells were killed. D, Decreased ATPase activity in *p*^0^MHS cells. ATPase activities were determined as in Materials and Methods. OLIGOM, Oligomycin (10 μM), Aurov B, Aurovertin B (60 μM). Data are means ± SEM of 3 experiments run in duplicates. *p < 0.05, **p < 0.01; ***p < 0.001.

Mitochondrial DNA (mtDNA) depleted (*ρ*^0^) cells lack mtDNA-encoded ATP synthase subunit F_0_6 and subunit F_0_8, and therefore they possess low ATPase activity.^38^,^39^ Using *ρ*^0^MH-S cells, we studied changes in mΔΨ, ATP levels, ATPase activity, and lifespan after LT addition. As shown in Figure 7A, the mΔΨ of *ρ*^0^MH-S was approximately 100-times lower than MH-S cells in accordance with the decreased proton pumping by respiratory complexes and lack of F_0_6 and F_0_8 in complex V. In response to LT *ρ*^0^MH-S decreased mΔΨ, while MH-S cells lost it (Fig. 7A). In a representative experiment (shown in Fig. 7B) less then 10% of *ρ*^0^MH-S cells showed Annexin V reactivity, while ∼95% of corresponding MH-S cells were killed 120 min after LT addition. LT-treated *ρ*^0^MH-S cells showed a significant (p = 0.01) loss of viability from 300 min onwards. Importantly, MH-S cells were killed by a sudden type of cell death (Fig. 7B, inset b), while *ρ*^0^MH-S cells showed distinct populations of cells that only bind Annexin V and had loss of membrane integrity (bound Annexin V, incorporate 7-AAD; Fig. 7B, inset c).

ATP levels in *ρ*^0^MH-S cells were 77% ± 6.7% of the corresponding control MH-S cells (Fig. 7C). At 90 min after LT addition, cellular ATP levels significantly decreased in MH-S cell cultures (Fig. 2A), while in *ρ*^0^MH-S cells ATP levels were intact (Fig. 7C). ATP levels showed a significant (p =0.05) decrease from 240 min on in *ρ*^0^MH-S cultures (Fig. 7C). These observations may be explained by the absence of mtDNA encoded F_0_6 and F_0_8 subunits of complex V. In support of these results, ATPase activity of *ρ*^0^MH-S cells was 17% ± 4.7% of the corresponding controls (Fig. 7D). Further, while LT-increased ATPase activity was 3-fold by 90 min in MH-S cells (Fig. 7D), we observed a ∼2-fold increase in ATPase activity at 240 min in *ρ*^0^MH-S. It is interesting to note that ATPase activity of *ρ*^0^MH-S cells can be inhibited only by aurovertin, while the corresponding MHS cells responded to both oligomycin and aurovertin (Fig. 7D).

## Discussion

LT exposure of susceptible macrophages results in rapid cytolysis called pyroptosis,^40^ a cell death type sharing characteristics of apoptosis and oncosis/necrosis.^41^ Due to the pivotal role of intracellular ATP level in the decision-making processes among types of cell death,^42^–^45^ we investigated its role in cytolysis of LT-treated susceptible macrophages. Here we show that death of LT-treated MH-S cells was tightly associated with ATP depletion caused by increased ATPase activity of F_1_F_0_ complex in mitochondria. The *p*^0^MH-S cells, in which mitochondrial ATPase activity is impaired, showed increased resistance to LT. These results suggest that inhibition of LF-triggered ATP depletion may protect macrophages from pyroptotic processes.

Opening of MPTP, dissipation of mΔΨ, and depletion of ATP were observed as early processes in cell death of LT-exposed MH-S macrophages, but there were no changes in intracellular ROS levels. The early events were followed by rapid morphological changes including membrane vesicle formation, swelling and plasma membrane rupture. The phosphatidyl serine rearrangement (annexin V binding) and simultaneous loss of membrane integrity (7AAD uptake) appeared in every toxin-exposed cell culture and only the start points of the events depended on LT’s concentration.

NALP1 is primarily responsible for macrophage susceptibility to toxin *via* inflammasome formation and activation;^4^ however, recent data indicate that inflammasome formation is a contributing, but not initiating, event in LT-mediated cytotoxicity and that earlier LT-mediated events leading to ion fluxes are required for death.^46^ The exact mechanism by which LF activates NALP1 is unknown, although intracellular K^+^ efflux was shown to be an important and specific trigger for inflammasome activation.^47^ A previous study reported an increase in membrane permeability to K^+^ together with a rapid conversion of ATP to ADP as early events after LT exposure.^27^ The authors concluded that K^+^ efflux would be expected to cause depletion of ATP *via* increased activity of Na^+^/K^+^ pumps. However, in our experiments ouabain, an inhibitor of Na^+^/K^+^-ATPase, did not prevent ATP loss and did not cause an increase in the lifespan of LT-treated cells. On the other hand, Na^+^/K^+^-ATPase is inhibited during hypoxia,^48^ thus hypoxia-like intracellular redox conditions (sub-physiological ROS levels) after LT addition, further diminished the likelihood of activation of Na^+^/K^+^-ATPase. Importantly, ATP depletion after LT exposure was observed at times and doses where the plasma membrane was still intact. Based on our results, we suppose that K^+^ efflux is a consequence of metabolic stress in LT-treated cells. In support, depletion of cellular ATP stores stimulates release of K^+^ through opening of ion channels in the plasma membrane of many cell types.^49^–^51^ Regulated release of K^+^ ions serves to minimize cellular injury during ATP depletion and protein kinase C is selectively involved in this mechanism.^50^ Indeed, activation of protein kinase C is required for mediating LT cytotoxicity.^52^ It has been shown that a decrease in cellular ATP levels is directly linked to membrane perturbation, and high KCl concentration partially prevents this event and increases survival of LT-treated cells.^10^ This effect of KCl could be associated with a block of membrane perturbation^10^ and also with inhibition of mitochondrial ATPase activity of macrophages.^53^ In our system, there was no ATP released into the medium of LT-exposed cells and we did not observe PARP activation, an energetically very expensive process, which can lead to the depletion of cellular energy stores.^28^ These results suggest that the energy crisis was facilitated by some other means, which implicates mitochondria in the LT-mediated ATP depletion.

Using Far-Western analysis, we showed that LF interacts with proteins of the F_1_F_0_ complex (subunit γ and β polypeptides) in both susceptible and resistant cells *in vitro*. These results were confirmed by immunoprecipitation of mitochondrial lysates from LT-treated MH-S cells and LA-4 cells. Interactions between LF and F_1_F_0_ subunit γ and β polypeptides were found in mitochondrial lysates of MH-S cells, but not in those of LA-4 cells. These observations were consistent with the absence of ATP depletion in LA-4 cells and raised the possibility that interactions of LF with F_1_F_0_ subunit proteins in susceptible cells lead to increased ATPase activity, which is the key event in LT’s cellular pathogenesis. Indeed, oligomycin, a nonselective F_1_F_0_-ATPase inhibitor,^36^,^37^,^54^ delayed ATP depletion and increased the survival of LT-treated cells.

F_1_F_0_ complex γ and β polypeptide chains are important components directing the flow of protons through the F_1_F_0_ complex for ATP synthesis. These polypeptides also regulate ATPase activity of the F_1_F_0_ complex.^31^,^32^,^55^ The catalytic turnover rate (>300 s^−1^) of ATP hydrolysis *via* the F_1_F_0_-ATPase is the greatest of any known ATPase.^31^,^32^ In theory, by interacting with γ and β polypeptides, LF may interfere with conformational changes and/or other steps in processes taking place in complex V during ATP synthesis thereby increasing its ATPase activity.^31^,^32^,^55^

Mitochondrial DNA-depleted (*p*^0^) cells lack F_0_6 and F_0_8, which are required for ATPase activity of F_1_F_0_ complex.^38^,^39^,^56^ Indeed, *p*^0^MH-S cells showed significantly lower ATPase activity and consequently less mΔΨ. These results are consistent with previous observations showing that a functional adenine nucleotide carrier and ATP were essential to generate mΔΨ in *ρ*^0^ cells.^56^ LT-induced ATP depletion in *p*^0^MH-S cells was significantly delayed, resulting in increased cellular lifespan.

Direct protein-protein interaction between LF and subunits of F_1_F_0_ complex detected by immunoprecipitation raises the question why LF does not perturb the F_1_F_0_ complex activity in bacterial cells. It is known that the sequence of eukaryotic subunit β is significantly longer and contains a shorter dimerization domain than that of the bacterial protein.^31^ It was also shown that the dimerization domain of mitochondrial subunit β is necessary for interaction with other proteins.^31^,^32^ Thus, differences in amino acid sequences between mammalian and bacterial β polypeptides explain the lack of LF inhibitory action on bacterial F_1_F_0_ complex.

LF, as a highly specific zinc-dependent metalloprotease, cleaves a series of kinases of the MKK family. In a recent study, a phage display system was used to investigate the substrate specificity of LF.^58^ Those peptide substrates that were capable of effective binding to LF carried a certain motif containing the same basic amino acid residues (mostly arginines) at p5-p4 positions and a branched hydrophobic amino acid residue at position 3.^58^ Searching the NCBI Protein database, we found that human mitochondrial F_1_F_0_ complex β (accession #: AAH16512) and γ (AAH20824) subunits, as well as mouse γ (NP_001106209) contain this motif close to their N termini. Taken together, it is possible that LF not only binds but also cleaves the polypeptides of F_1_F_0_ ATPase complex. We plan to analyze whether the protease activity of LF is relevant to its ability to increase F_1_F_0_ complex ATPase activity.

LT macrophage killing is an inflammasome-mediated event, which requires NALP1-mediated caspase-1 activation. A basic question is how ATP depletion is involved in these processes. We hypothesized a model in which ATP depletion *via* increased mitochondrial F_1_F_0_-ATPase activity triggers intracellular K^+^ efflux, which activates late events including NALP1-inflammasome formation and caspase-1 activation to execute cell death. In support, *p*^0^MH-S cells, in which NALP1-caspase-1 system is intact but ATPase activity is low, show increased resistance to LT. However, further experiments are needed to confirm our proposed model.

The current principal treatment for various forms of anthrax infection is antibiotics.^59^ Because of the increased frequency and growing threat of antibiotic-resistant strains, the need for new therapeutic agents other than antibiotics is vital. Our results offer the promise that pharmacological inhibition of LF’s mitochondrial targeting or interaction with the F_1_F_0_ complex and/or inhibition of F_1_F_0_ ATPase could be effective. At the cellular level, such an intervention may increase the lifespan of macrophages *in vivo*, which could provide time for development of adaptive immunity to tackle the pathogen.

## Figures and Tables

**Table 1:** LF-interacting mitochondrial proteins.

Gel band ID (Fig. 6C)	1Protein	Theoretical mass (kD)	Swiss-Prot ID	Score (bits)/E value	Sequence coverage 2MS
A1	3Mitochodrial ATP synthase H^+^ transporting, F_1_ subunit beta	56.3	P56480	36.3/0.015	N.D.
A2	Mitochodrial ATP synthase subunit alpha (precursor)	59.7	Q03265	40.1/0.010	N.D.
B	3Mitochodrial ATP synthase H^+^ transporting F_1_ complex gamma polypeptide	30.3	Q8C2Q8	39.7/0.014	N.D.
C1	Mitochodrial ATP synthase subunit O (precursor)	23.4	Q9DB20	42.2/0.002	N.D.
C2	Mitochodrial ATP synthase H^+^ transporting F_0_ complex subunit b, isoform 1 (precursor)	28.9	QSI0W0	35.8/0.200	N.D.
D	Mitochodrial cytochrome bc-1 complex subunit core 1 (precursor)	52.8	Q9CZ13	39.2/0.019	N.D.
E	Mitochodrial cytochrome bc-1 complex subunit core 2 (precursor)	48.2	Q9DB77	N.D.	4.3 × 10^−46^
F	Heat shock protein (60 kD, mitochondrial)	60.9	P63038	38.8/0.025	1.4 × 10^−42^
G	Mitochondrial trifunctinal enzyme subunit beta (precursor)	51.6	Q99JY0	N.D.	1.7 × 10^−25^
H	3-oxoacid CoA transferase	56.0	Q3UK61	41.8/0.003	N.D.
I	Mitochondrial NADH dehydrogenase flavoprotein 1 (precursor)	50.8	Q91YT0	14.6/0.18	N.D.
J	Short-chain specific acyl-CoA dehydrogenase (precursor)	45.2	Q07417	N.D.	2.2 × 10^−35^

1 Proteins were identified by N-terminal microsequence analyses using Applied Biosystems’ cLC 494 Protein Sequencer;

2 N-terminal blocked proteins were identified by MALDI-TOF/TOF/MS (Applied Biosystems’ 4800);

3 Confirmed by Western blot analyses (Materials and Methods) N.D, not done.
